# Impact of an addiction medicine consult team intervention in a Canadian inner city hospital on acute care utilization: a pragmatic quasi-experimental study

**DOI:** 10.1186/s13011-022-00445-7

**Published:** 2022-03-12

**Authors:** Ginetta Salvalaggio, Kathryn A. Dong, Elaine Hyshka, Christopher McCabe, Lara Nixon, Rhonda J. Rosychuk, Klaudia Dmitrienko, Judith Krajnak, Kelly Mrklas, T. Cameron Wild

**Affiliations:** 1 Inner City Health and Wellness Program, Royal Alexandra Hospital, Edmonton, AB Canada; 2 Faculty of Medicine and Dentistry, University of Alberta, Edmonton, AB Canada; 3 School of Public Health, University of Alberta, Edmonton, AB Canada; 4 Institute of Health Economics, Edmonton, AB Canada; 5 Cumming School of Medicine, University of Calgary, Calgary, AB Canada; 6 Primary Health Care Program, Alberta Health Services, Edmonton, AB Canada; 7 Strategic Clinical Networks, Provincial Clinical Excellence, Alberta Health Services, Calgary, AB Canada

**Keywords:** Substance-related disorders, Vulnerable populations, Health services, urban, Patient care team

## Abstract

**Background:**

Inner city patients have a higher illness burden and need for care, but experience more unmet care needs. Hospital Addiction Medicine Consult Teams (AMCTs) are a promising emerging intervention. The objective of this study was to assess the impact of a Canadian AMCT-like intervention for inner city patients on reduction in high emergency department (ED) use, hospital admission, and inpatient length of stay.

**Methods:**

Using a community-engaged, two-arm, pre-post, longitudinal quasi-experimental study design, 572 patients reporting active substance use, unstable housing, unstable income, or a combination thereof (302 at intervention site, 270 at control sites) were enrolled. Survey and administrative health service data were collected at baseline, six months post-enrolment, and 12 months post-enrolment. Multivariable regression models tested the intervention effect, adjusting for clinically important covariables (inpatient status at enrolment, medical complexity, age, gender, Indigenous identity, shelter use, opioid use).

**Results:**

Initial bivariable analyses demonstrated an intervention effect on reduction in admissions and length of stay, however, this effect was no longer significant after adjusting for covariables. There was no evidence of reduction in high ED use on either bivariable or subsequent multivariable analysis.

**Conclusions:**

After adjusting for covariables, no AMCT intervention effect was detected for reduction in high ED use, inpatient admission, or hospital length of stay. Further research is recommended to assess other patient-oriented intervention outcomes.

**Supplementary Information:**

The online version contains supplementary material available at 10.1186/s13011-022-00445-7.

## Background

Substance use is a growing threat to public health. An estimated 19% of Canadians exceed low risk drinking guidelines [1, 2], and 22% meet criteria for one or more substance use disorders (SUDs) in their lifetime (4% meet criteria in the past 12 months) [3]. Mortality attributable to substance use has risen dramatically since 2016 [4], and problematic substance use is associated with infectious disease, trauma, mental illness, and other chronic diseases [5–7]. In 2015–16 there were 77,000 Canadian hospitalizations attributable to alcohol [8] and currently over 400 Canadians are hospitalized daily due to harms caused by alcohol and other drugs [9]. Among adults age 18–64, SUDs were the second most common cause of inpatient hospitalization in Canada in 2019–20, exceeding all other causes of hospitalization with the exception of giving birth [10].

Deleterious substance-related health outcomes are more common among people living in adverse socioeconomic circumstances [11–16]. Concurrent poverty and substance use is particularly common in major urban centres [17, 18]. For example, 235,000 Canadians experience homelessness in a given year, the majority of whom are in urban centres [19]; and of these, a large proportion report alcohol or other drug use along with mental health concerns [18]. Low income, low employment, repeated exposure to violence, and the experience of racism and systemic discrimination are other commonly cited adverse circumstances [20–22]. Lack of government-issued identification documents, inconsistent medication coverage or other health benefits, as well as inconsistent transportation or social supports all create barriers to accessing healthcare and other services [23, 24]. These socioeconomic barriers to timely, community-based care are reflected in the association between low socioeconomic position and more frequent ED use and hospitalization [25, 26].

Despite their higher burden of illness and need for care, health systems are ill-equipped to provide care to people with co-occurring adverse socioeconomic circumstances and substance use challenges. Inner city patients receive less primary and preventive care than other patients [27, 28]. Moreover, they report multiple unmet care needs including medication, information, skills training, harm reduction supports, and assistance with social issues [29–31]. A recent survey of Canadian inner city emergency department (ED) patients experiencing unstable housing and/or problematic substance use suggested that they would like to access wraparound supports beyond treatment of their acute medical condition, such as social stabilization and addiction stabilization, while accessing acute care [32].

Although community-based care plays an essential role in supporting the health of inner city populations, consistent access is a challenge, and illness severity may require hospital assessment more often than for general populations. As one of few available 24/7 points of access to care, hospitals present an opportunity to address multiple unmet care needs for people who use substances [11, 18]. However, due to expectations of abstinence and/or suboptimal SUD management, hospitals can also be a high-risk, relatively hostile environment for patients who use substances resulting in premature departure, untreated health problems, and unplanned readmission [33, 34].

Addiction Medicine Consult Teams (AMCTs) are a newly emerging approach to improving hospital care for patients with SUDs. These multidisciplinary consult teams work with attending physicians and unit staff to provide rapid access to addiction treatment for hospitalized patients and connection to ongoing treatment and wrap-around supports post-discharge. Research regarding the impact of this emerging response to the needs of hospitalized patients with SUDs is limited but promising: while there are no published randomized controlled trials measuring the efficacy of AMCTs, there is growing evidence outlining their impact. AMCTs successfully engage patients in addiction treatment [35] and this hospital-based, patient-centred SUD care is associated with reduced substance use and mortality [36, 37]. Evidence to date from the United States indicate that AMCTs in that setting can reduce inpatient length of stay for patients with SUDs, reduce 30-day unplanned readmission rates, and increase uptake into outpatient addiction treatment [38–40]. Patient-reported benefits of the AMCT model include: improved care experiences, reduced substance use, better mental and emotional well-being, and in some cases, enhanced socio-economic conditions [41]. Beyond these positive clinical outcomes and patient benefits, AMCTs also positively influence the knowledge, attitudes, and experiences of care providers [37, 42, 43] further enhancing patient engagement and the development of care relationships.

Although the elements of AMCT-type interventions are evidence-informed from community settings, multi-component hospital interventions for people who use substances are comparatively novel, and evidence to guide implementation outside of the United States setting is limited. In light of the positive evidence generated to date from observational studies, controlled longitudinal research regarding AMCT-type interventions is warranted [33–41]. Moreover, the role of enhanced, whole person approaches to the AMCT model for people experiencing both adverse socioeconomic circumstances and SUDs has not been examined. Thus, the objective of this study was to determine whether an AMCT intervention implemented among inner city patients positively influenced acute healthcare utilization outcomes, specifically reduction in ED use, hospital admissions, and inpatient length of stay. We hypothesized a clinically significant intervention effect of a 20% reduction in high ED use, our primary outcome.

## Methods

### Overview and Study Design

The Enhanced Multidisciplinary Care for Inner City Patients Seeking Acute Health Care (EMCIH) study used a two-arm, pre-post, longitudinal quasi-experimental study design; details are described in our published protocol [44]. A pragmatic, non-randomized design was chosen because individual-level randomization was not feasible for a site-level service innovation, and insufficient comparable and implementation-ready sites existed within the same Canadian province to permit site-level randomization. A community-engaged research approach was used throughout the study period; the study protocol, implementation, and interpretation received iterative guidance from a Community Advisory Group of people with lived experience of both substance use and the acute healthcare system.

### Participants

Intervention arm participants were recruited from among inpatients and outpatients within two weeks of their initial assessment by the intervention site’s AMCT in Edmonton, Canada. Consult requests to the AMCT were initiated by the patients’ most responsible physician and could come from any inpatient service or the emergency department (ED). During the recruitment period, 611/1448 (42%) of AMCT referral requests originated from the internal medicine service, 422 (29%) from the ED, 162 (11%) from surgery, 91 (6%) from the hospitalist team, and the remainder from a variety of other specialty services. AMCT referral criteria were active substance use related concerns and/or social instability. These more inclusive intervention eligibility criteria were applied due to a large overlap in substance use and unstable housing in the target population based on intervention site clinical experience, and a lack of reliable pre-study data on which hospitalizations would benefit from referral. Control arm participants were recruited from inpatients (medical, surgical, and psychiatric) and outpatients (emergency and urgent care) seen in two acute care facilities serving a similarly disadvantaged patient population in the nearby city of Calgary, Canada. Potential participants were identified by treating clinicians. In both study arms, research assistants confirmed study eligibility (problematic substance use, unstable housing, and/or unstable income), explained the study, obtained informed consent for the overall study, and obtained additional consent for retrieval and linkage of administrative health service data to primary survey data. Research assistants then completed a detailed contact information sheet detailing both traditional and non-traditional means of contact (e.g. name of outreach worker, usual places frequented) to facilitate participant follow-up.

### Intervention

The intervention site AMCT was launched in July 2014, in response to patient-voiced unmet care needs. The intervention development team considered substance-use related care, health promotion, and interventions addressing the social determinants of health to be equally important. Feedback from our Community Advisory Group, and later from our parallel process evaluation, confirmed the importance of whole person care in acute health service delivery and the need to incorporate this approach into the AMCT model. The resulting multidisciplinary consultation service was co-designed with patients, clinicians, and other stakeholders. At study inception, the core clinician complement included rotating physicians with addiction medicine expertise (one physician available 0800 h–2100 h, seven days/week), a nurse practitioner, and a social worker; an addiction counselor and peer support worker were added in the latter half of the study period. Emphasizing bridging support to promote care continuity during the hospital-community care transition, the AMCT provided management of intoxication, withdrawal, and/or acute pain; initiation or continuation of opioid agonist treatment; referral to addiction treatment and recovery programs; overdose prevention interventions; screening for sexually transmitted and blood borne infections; and, social stabilization supports including assistance with housing, income, and/or obtaining healthcare coverage, photo identification or health care card. The AMCT provided consultation on referral by the attending clinical team to both hospital inpatients and ED patients, the latter of whom were offered follow-up assessment in a transitional clinic on site. The intensity, duration, and specific components offered were tailored to patient needs and preferences. Usual care at the control sites consisted of assessment and treatment by an attending team without specific expertise in addiction medicine or related individualized care plans to manage the unique health and social needs of patients who use substances; attending teams could access a nurse-clinician on select days for addiction medicine advice.

### Data Collection

Participants completed a baseline quantitative survey administered by a research assistant. Survey variables (see supplementary file 1) included sociodemographic information (e.g. gender, ethnicity, housing status), substance use behaviour (e.g. alcohol and drug [45, 46] consumption patterns, experience of overdose), perceived health (EQ-5D [47]), and unmet need for care (Perceived Needs for Care Questionnaire [48]). Although follow-up survey data were collected for the purposes of secondary outcome analysis, only baseline survey data were used for the analyses presented herein.

The research team collaborated with the provincial administrative health service data custodian to retrieve and link administrative data for consenting participants. Data on healthcare encounters were retrieved for a 540-day period including 180 days prior to and 360 days following the baseline study enrolment date for each participant.

### Outcome measures

Main outcome measures were obtained from administrative health data. The primary outcome measure was reduction in high ED use, where the baseline measurement period was defined as 180 days prior to baseline study enrolment date, and the follow-up measurement period was defined as 180 days falling between six and 12 months after the baseline study enrolment date. Consistent with other studies of complex populations with high acute case use, “high ED use” was defined as more than two visits to either an ED or urgent care (UC) facility during either 180-day period [49, 50]. We considered using ED frequency counts across the 540-day study period, but decided to create a change variable omitting the 180-day period immediately after intervention start because we anticipated that 1) frequency of ED visits would be non-normally distributed [26, 51] and 2) ED visits would increase as a function of the acute care-based intervention before subsequently decreasing.

Secondary acute care utilization outcome measures included reduction in inpatient admissions and reduction in hospital length of stay, calculated using the same 180-day pre- and post-periods used for the primary outcome measure.

Covariables were identified *a priori* based on their potential to exert clinically meaningful effects on the intervention and/or outcome. Most covariables were collected via the baseline participant survey. These included age, gender (male, female, transgender), Indigenous ethnicity (coded as Indigenous if responses of “First Nations”, “Métis”, “Inuit”, “Cree”, “Aboriginal”, “Non-status Indian”, or “Treaty” were given to the question “What ethnic group do you identify with?”), and unmet need for care (as measured by the Perceived Needs for Care Questionnaire) [48]. Shelter use was recorded as present if a participant reported sleeping in a hostel or shelter in the previous six months; it was chosen as our indicator of housing instability as the most common sleeping arrangement for those without housing. Opioid use was recorded as present if any non-prescribed use of intravenous or non-intravenous opioids was reported in the previous six months. Two non-survey covariables were also included. Clinical Risk Group (CRG [52], a diagnosis-based indicator of medical complexity that takes into account disease chronicity and severity and its expected impact on morbidity, mortality, and future service needs) was retrieved from the recruitment hospital admission or ED/UC visit and was coded low, medium or high risk. Recruitment location was documented by research staff as inpatient (ward) or outpatient (ED, UC).

### Data Analysis

We hypothesized a clinically significant intervention effect of a 20% reduction in high ED use, which a power analysis indicated would require 103 participants per study arm for detection (two-sample test of proportions, α = 0.05, power = 80%). We anticipated 50% study attrition as well as administrative data validation challenges (consistent with studies of similar cohorts [53, 54]), and therefore set a recruitment target of 300 for each study arm.

Generalized linear mixed models were used for analysis. The unit of analysis was the participant and all analyses were performed in R [55]. Summary statistics (e.g., frequencies, percentages, means, standard deviation [SD], median, interquartile range [IQR] represented as 25th percentile, 75th percentile) described covariables and outcomes by study arm. Initially, two-sample tests of proportions, chi-square tests, and t-tests compared outcomes by study arm and covariables. This was followed by multiple logistic regression analyses to compare outcomes across study arms, adjusted for age, self-identified gender, Indigenous status, housing status, opioid use, unmet need for care, CRG category, and recruitment location. Given the small number of participants that identified as transgender (n = 2), transgender was combined with the female category in all regression analyses. As CRG had 11 missing values, missing values were assumed to be missing at random and five imputed datasets using the multivariate imputation by chained equations (MICE [56]) package were created and regression models pooled. Odds ratios (ORs) and associated 95% confidence intervals (CIs) are presented and a *p*-value less that 0.05 was considered statistically significant.

## Results

### Sample description

**Fig. 1:**
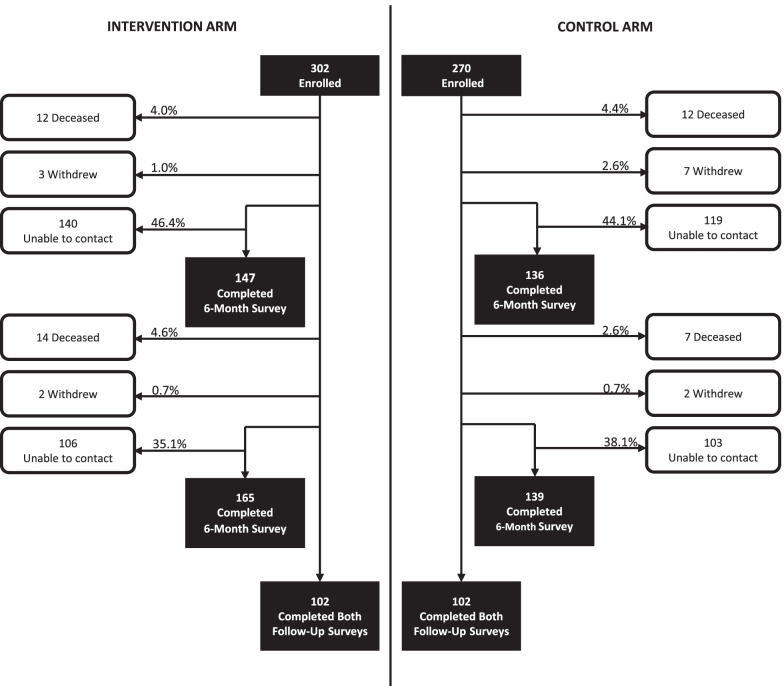
Participant flow diagram – follow up survey completion

**Fig. 2:**
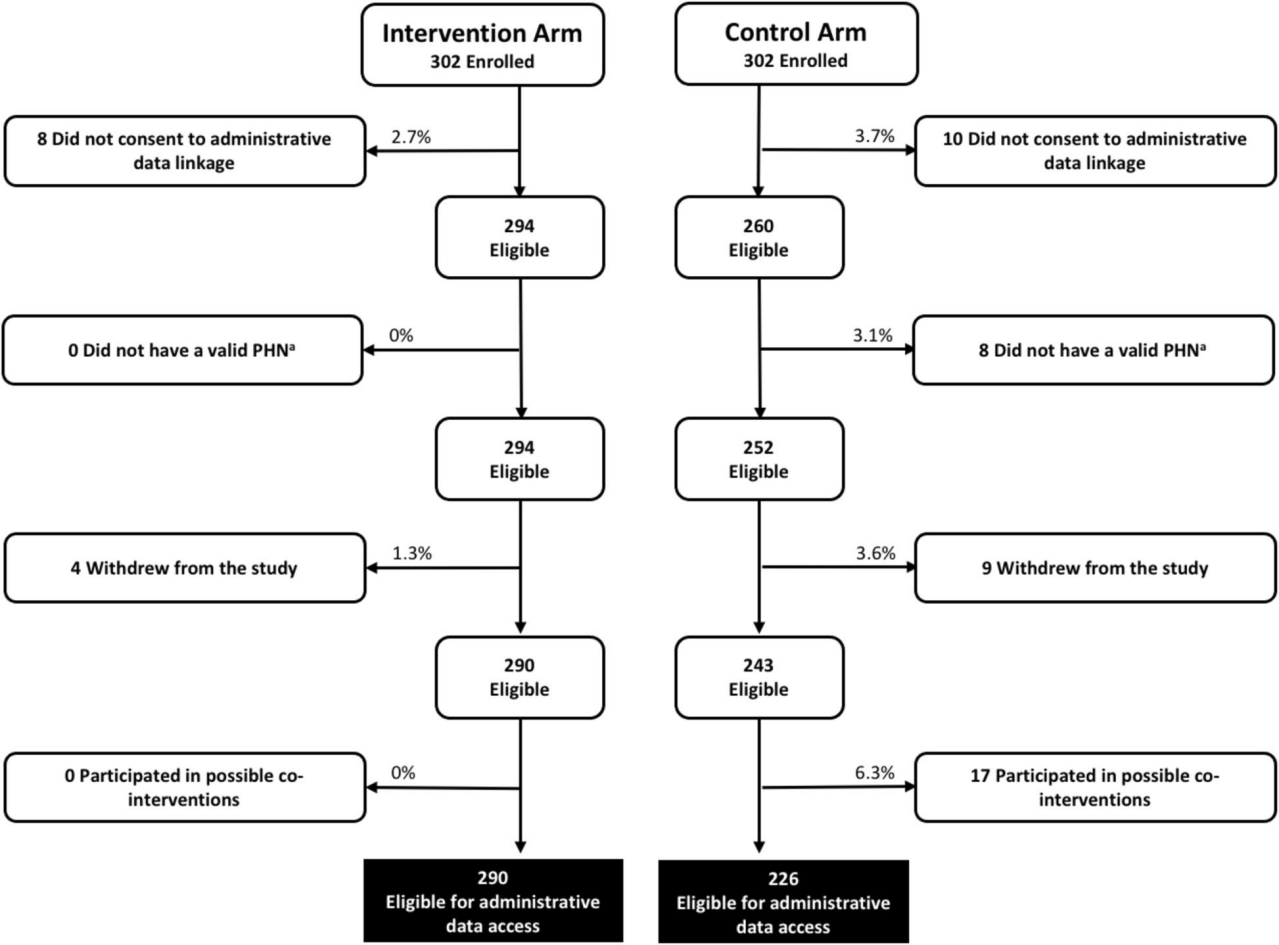
Participant flow diagram – administrative data access

A total of 572 patients (302 intervention arm, 270 control arm) were enrolled into the study and completed baseline survey data collection between August 2014 and June 2016 (Fig. 1). At the time of recruitment over 90% of the sample reported recent alcohol or substance use, and over 70% reported unstable housing, unstable income, or both (Table 1). Almost all participants (*n* = 554; 97%) consented to the retrieval and linkage of their longitudinal administrative health services data. After removing invalid personal healthcare identifiers, identifying study withdrawals, and a small number of control arm participants potentially exposed to local case management co-interventions, the final sample included 516 participants (290 intervention arm, 226 control arm) for analysis (Fig. 2). Attrition due to death, study withdrawal, or inability to contact was similar across both study arms. There were no significant deviations from the original study protocol throughout the data collection period.

**Table 1:** Participant characteristics by study arm

Patient Characteristic	Intervention (*N* = 290)	Control (*N* = 226)	Significance (***p***-value)
Age (mean)	44 years	45 years	0.64
Male	177 (61%)	154 (68%)	0.12
Indigenous	121 (42%)	54 (24%)	<0.01*
Shelter use (past 6 months)	71 (26%)	113 (50%)	<0.01*
Opioid use (past 6 months)	111 (38%)	60 (27%)	0.01*
Perceived unmet need for care^a^	212 (73.1%)	184 (81.4%)	0.04*
Clinical Risk Group^b^			
High	137 (47%)	67 (30%)	
Medium	94 (32%)	73 (32%)	
Low	54 (19%)	80 (35%)	
Recruitment Location (Inpatient)	245 (86%)	112 (50%)	<0.01*
Participant Eligibility			
Unstable housing^c^	209 (72%)	175 (77%)	
Alcohol/drug use (past 6 months)^d^	272 (94%)	205 (91%)	
Unstable income^e^	208 (72%)	184 (81)%	

^*^Statistically significant at the level of 0.05

^a^As measured by the Perceived Need for Care Questionnaire [42]

^b^Clinical Risk group scores; low = 1–3, medium = 4–5 and high = 6–9

^c^As measured by the baseline survey (see supplementary file 1). Participants met this eligibility criteria if they reported their living situation as unstable, had no home or slept in more than 5 places in the past 6 months

^d^As measured by the baseline survey (see supplementary file 1). Participants met this eligibility criteria if they consumed any alcohol or injection or non-injection drugs in the past 6 months

^e^As measured by the baseline survey (see supplementary file 1). Participants met this eligibility criteria if they reported their income below the low income level cut-off

Study arms differed at baseline across a number of covariables (Table 1). More intervention arm participants used opioids and were Indigenous compared to control arm participants; whereas fewer intervention arm participants had used a shelter in the past six months. Intervention arm participants were more likely to be recruited as inpatients, and also had a higher level of medical complexity, as defined by the CRG.

### Acute Care Outcomes

With respect to the primary outcome, 28.3% (82/290) of the intervention arm participants experienced a reduction in high ED use compared to 33.6% (76/226) in the control arm (Table 2, *p* = 0.225). There was no difference between study arms in the proportion of participants experiencing a reduction in high ED use. When these bivariate results were adjusted by covariables in a logistic regression analysis, study arm remained non-significant and only CRG predicted the primary outcome (Table 3). During preliminary analysis we explored additional modeling using an ED visit count variable rather than a change variable, however, the result of this analysis were similar and thus are not included herein.

**Table 2:** Outcomes by study arm

	Intervention (*N* = 290)	Control (*N* = 226)	Significance (***p***-value)
Reduction in high emergency department use	82 (28.3%)	76 (33.6%)	0.225
Reduction in hospital admissions	212 (73.1%)	132 (58.4%)	0.001*
Reduction in hospital length of stay	212 (73.1%)	138 (61.1%)	0.005*

*Statistically significant at the level of 0.05.

**Table 3:** Regression modeling results for each study outcome

Predictor Variable	Reduction in High EDc Use		Reduction in Inpatient Admissions		Reduction in Hospital LOSd	
	Adjusted OR [95% CI]	*P*-Value	Adjusted OR [95% CI]	*P*-Value	Adjusted OR [95% CI]	*P*-Value
Study Arm	0.74 [0.48,1.15]	0.183	0.89 [0.55,1.45]	0.633	0.64 [0.38,1.08]	0.093
Agea	0.99 [0.97,1.01]	0.165	0.99 [0.97,1.01]	0.197	0.98 [0.96,1.00]	0.025*
Male gender	0.99 [0.65,1.49]	0.941	1.18 [0.75,1.84]	0.479	1.23 [0.77,1.97]	0.374
Indigenous identity	1.16 [0.75,1.77]	0.507	0.75 [0.47,1.19]	0.224	0.75 [0.46,1.22]	0.252
Shelter use	1.46 [0.94,2.24]	0.090	1.23 [0.75,2.01]	0.410	1.12 [0.68,1.85]	0.665
Opioid use	1.24 [0.80,1.92]	0.334	1.65 [1.00,2.74]	0.052	1.42 [0.84,2.39]	0.193
Had unmet care needs	1.05 [0.65,1.69]	0.840	1.03 [0.62,1.71]	0.908	0.90 [0.53,1.53]	0.708
Low CRGb	0.54 [0.32,0.93]	0.026*	0.89 [0.51,1.54]	0.676	0.86 [0.49,1.53]	0.617
Medium CRG	0.79 [0.49,1.27]	0.334	1.14 [0.68,1.91]	0.610	1.31 [0.76,2.23]	0.361
Outpatient recruitment	1.06 [0.66,1.71]	0.799	0.10 [0.06,0.17]	<0.001*	0.07 [0.04,0.112]	<0.001*

*Statistically significant at the level of 0.05

^a^OR is per year of age

^b^*CRG* Clinical Risk Group; low and medium CRG are in reference to high CRG

^c^*ED* Emergency Department

^d^*LOS* Length of Stay

For secondary outcomes, bivariate results indicated that pre- to post-test reductions in inpatient admissions were more common among participants in the intervention arm (212/290, 73.1%, Table 2) compared to control arm participants (132/226, 58.4%, *p* = 0.001). However, in subsequent multivariable analyses adjusted by covariables (Table 3), study arm was no longer statistically significant, and recruitment location and baseline opioid use were the variables associated with this outcome.

Finally, more intervention arm participants exhibited reductions in the inpatient length of stay (212/290, 73.1%, Table 2) than control arm participants (138/226, 61.1%; *p* = 0.005). When adjusted by study covariables (Table 3), study arm was no longer statistically significant and age and recruitment location were the variables most associated with outcome.

Because of baseline site-level differences between the study arms, the research team and administrative data partners created a second geographic comparison group at the intervention site, using a diagnosis-based sampling frame definition developed for the study. Intervention site controls were matched with intervention recipients according to available variables of date and admission status (inpatient vs outpatient) of index encounter, complexity (CRG), age, and sex. Healthcare utilization measures retrieved for this additional cohort included ED use, inpatient admissions, and length of stay. Patterns of acute care use for the internal matched controls did not differ from those observed among the formal study control arm. Nevertheless, there may be important macroeconomic and/or healthcare throughput differences between the two cities in this study that may have influenced our results.

## Discussion

Though preliminary bivariable analyses from this quasi-experimental comparison of an AMCT intervention to usual care for inner city patients suggested that the intervention reduced acute care utilization, full modeling with inclusion of covariables did not provide statistically significant evidence of an intervention effect. These multivariable analyses indicated that illness complexity and severity – specifically CRG and outpatient recruitment status– accounted for the differences observed across study arms. Our findings contrast with some studies of AMCT interventions [38–40] but align with other studies [57]. In the studies demonstrating an intervention effect, one focussed on patients with opioid use disorder specifically where opioid agonist therapy initiation could be expected to confer significant stabilization and benefit in terms of acute care use [36]. Another included universal screening for substance use disorder which may have identified patients with less severe, and more easily treated addiction [37]. Variable intervention effects observed in the literature could also suggest that such interventions may be beneficial but insufficient in reducing acute care utilization without addressing intersecting system barriers; conversely, positive findings elsewhere may reflect the influence of unmeasured confounding factors. Identifying whether an intervention for complex needs works is particularly challenging, given that local contextual factors such as housing availability, toxicity of drug supply, and community-based supports contribute to variance in acute care utilization [13, 58]. Broader contextual factors such as health system organization (e.g. structure of, and access to care) and sociopolitical environment may also be contributing to the contrasting results between this Canadian study and many of the largely US-based prior AMCT studies.

Health systems are understandably concerned with making efficient use of resources, and there has been an increased system focus on reducing potentially avoidable ED visits, including the development of measures for tracking ED visits for Family Practice Sensitive Conditions (FPSC) [59]. In the EMCIH study sample, the proportion of patients with ED visits for FPSCs was small (16%), lower even than that for the general population (20%). Moreover, the relatively high mortality rate experienced by complex high needs populations [4] (including participants in both arms of this study) underscores their high illness burden and need for acute medical care. This suggests that most ED visits by study participants were unavoidable, and unlikely to change based on the availability of team-based primary care or hospital bridging supports. Given the lower level of health among inner city populations, and the likely ongoing need for periodic hospital-based care, health system leaders may find greater efficiency from addressing modifiable social determinants of health, improving care integration, and enhancing care continuity for inner city patients undergoing transitions between hospital and community-based care.

The length of follow-up period in this study may be considered relatively short for such a complex healthcare intervention evaluation. Indeed, many changes in healthcare utilization occur after more than 12 months for other complex interventions (e.g. patient centred medical home models) [60, 61]. However, there are a number of other patient-oriented outcomes to consider that could confer intervention benefit beyond acute healthcare utilization, and may be associated with a reduction in acute healthcare utilization patterns over the longer term. Rather than rapidly reducing acute care use, the complex team intervention may be better designed to effect changes in quality of life, unmet care needs, and visits to primary care providers, all of which have important healthcare utilization implications. The intervention also addressed several social determinants of health (e.g. housing, income support, medication coverage) and substance use which the EMCIH study was not primarily designed to assess. Finally, patient satisfaction [62]—a prominent theme in our parallel process evaluation [41]—is another outcome that may unintentionally have generated ED visit demand over the shorter term. Our qualitative research reported elsewhere [35] showed that patients receiving the intervention reported improved hospital care experiences involving the development of trust in the healthcare team. For patients who are conventionally underserved and discriminated against, the intervention team’s nonjudgmental and relationship-oriented approach may well have encouraged patients to seek needed care when they previously would have otherwise delayed or avoided it.

### Limitations

We employed a quasi-experimental study design for feasibility reasons; the null findings may in part be due to the lack of power inherent to this approach. Moreover, we detected baseline inequivalence in outcome measures. The multi-pronged complex intervention under study served multiple subgroups of patients (e.g. different primary substances of concern) with services tailored to individual needs. To address these differences at the individual participant level, we adjusted for observed baseline inequivalence across study arms and included clinically important covariables in the statistical models. However, a number of unmeasurable contextual factors are likely at play. We considered propensity weighting, but determined that this approach would not fully account for observed differences.

It is plausible that due to understandable limitations in the capacity of the newly formed intervention team, and benefits of the intervention perceived by referring clinicians, recruited participants were a group who not only met eligibility criteria but had a high level and acuity of need that stood out within eligible patients. This is reflected in the high level and acuity of need seen in the baseline questionnaire data and particularly high complexity (CRG) in the intervention group. Thus selection bias may have influenced our results.

The rollout of the EMCIH study at the control arm sites was met with excitement at the possibility of future intervention spread to other geographic regions within the same health system. Research staff on site were well received by staff and participants alike. A Hawthorne effect resulting from control arm participants responding favourably to their encounters with study and clinical staff following study enrolment cannot be ruled out; however, given the lack of support options available to participants beyond three structured quantitative data collection sessions and usual care, we believe a Hawthorne effect is unlikely to have exerted a significant enough influence to change acute care utilization.

The EMCIH study protocol occurred during the early intervention implementation period. The intervention has since undergone significant refinement (e.g. enhanced team composition, dedicated team expansion to the ED, addition of specific harm reduction supports such as managed alcohol programming, naloxone kit distribution, and a supervised consumption service) in response to referral patterns and consultation with program recipients and stakeholders. Intervention adaptation was also critical to address evolving population needs, most notably the advent of Canada’s opioid poisoning epidemic shortly after the launch of the intervention, and the subsequent prioritization of harm reduction and immediate access to opioid use disorder treatment options. The study findings reported herein reflect an earlier iteration of the intervention prior to intervention refinements having occurred. The team is engaged in ongoing program data collection and quality improvement work to assess the impact of such refinements.

## Conclusions

As originally conceived and implemented, an AMCT intervention was not associated with significant reductions in ED use, inpatient admission, or hospital length of stay. The relationships built between AMCT staff and patients may encourage patients to seek out care when they may otherwise have avoided it. The intervention continues to evolve in response to early study findings, stakeholder input, and emerging population needs. Further research is recommended to assess other patient-oriented intervention outcomes such as changes in the burden of unmet care needs, housing, income support, and substance use stabilization.

## Supplementary Information

**Additional file 1: Supplementary File 1.** Patient Outcome Evaluation Survey.

## Data Availability

The datasets generated and/or analysed during the current study are not publicly available due to data sharing agreements with participating administrative data custodians, but are available from the corresponding author on reasonable request.

## Abbreviations

AMCT: Addiction Medicine Consult Team
CI: Confidence Interval
CRG: Clinical Risk Group
ED: Emergency Department
EMCIH: Enhanced Multidisciplinary Care for Inner City Patients Seeking Acute Health Care
FPSC: Family Practice Sensitive Conditions
IQR: Interquartile Range
OR: Odds Ratio
SD: Standard Deviation
SUD: Substance Use Disorder
UC: Urgent Care
