# Supplementary material for: Impact of an addiction medicine consult team intervention in a Canadian inner city hospital on acute care utilization: a pragmatic quasi-experimental study
Source: Subst Abuse Treat Prev Policy. 2022 Mar 12;17:20. doi: 10.1186/s13011-022-00445-7 (PMC8917626; doi:10.1186/s13011-022-00445-7)
Supplement: Supplementary file 1 — Additional file 1: Supplementary File 1. Patient Outcome Evaluation Survey. [file 13011_2022_445_MOESM1_ESM.pdf]

PATIENT OUTCOME EVALUATION SURVEY
Version 6

Study Participant ID:

Chart Number:

Interview date: __ __/__ __/__ __ __ __
DD MM YEAR

Patient recruited from (unit or department): _____________

Interviewer initials: _____________

Interview start time: ____________ AM or PM (*please circle*)

Is the information collected in the interview significantly distorted by the participant's misrepresentation?

☐ No
☐ Yes
☐ Not sure

Is the information collected in the interview significantly distorted by the participant's inability to understand?

☐ No
☐ Yes
☐ Not sure

Thank you for contributing to this study. As you go through the survey, please keep in mind that there are no wrong answers. It's very important that you answer as honestly as you can. We realize some of these questions are sensitive. If you do not want to answer a question, you do not have to. It is better for you to refuse to answer a question than to give a false answer. We take your privacy very seriously. All the information that you provide will only be kept between you and me. We never report any individual information.

If there are any questions you don't understand, please stop me and ask for clarification. The interview takes about an hour. If you need a break, let me know and we can stop for a short rest before we finish the interview.

First of all we'd like to find out a little bit about you.

1. What is your age? ________________________ years

2. What is your gender?
   - ☐ Male
   - ☐ Female
   - ☐ Transgendered

3. What ethnic group do you identify with?
   - ☐ White
   - ☐ First Nations
   - ☐ Métis
   - ☐ Other

   If you identify with another ethnic group, please specify: ______________________________________

A few more questions about you:

4. What is the highest level of education that you have attained?
   - ☐ Some junior high school or less
   - ☐ Completed junior high school
   - ☐ Some high school
   - ☐ Completed high school
   - ☐ Some college/trade school
   - ☐ Completed college/trade school
   - ☐ Some undergraduate university
   - ☐ Completed undergraduate university
   - ☐ Some graduate university
   - ☐ Completed graduate university

5. Are you in a relationship right now?
   - ☐ Yes → **Go to Q6**
   - ☐ No → **Go to Q6a**

6. If yes, what type of relationship is it?
   - ☐ Legally married
   - ☐ Common-law
   - ☐ Boyfriend or girlfriend
   - ☐ No response
   - ☐ Other, please specify: ____

   a. Have you had any children?
      - ☐ Yes, how many? ______________________________________ → **Go to Q6b**
      - ☐ No → **Go to Q7**
      - ☐ Don't know → **Go to Q7**
      - ☐ No response → **Go to Q7**

b. Are they usually in your care (i.e., are you responsible for all or part of their day-to-day care)?

- ☐ Yes, how many? ____
- ☐ No
- ☐ Don't know
- ☐ No response

Now, I would like to ask you some questions about your living situation.

7. How long have you lived in this city?

- ☐ 0-5 years
- ☐ 6-10 years
- ☐ 11-15years
- ☐ 16-20 years
- ☐ 21-25 years
- ☐ 26-30 years
- ☐ Over 30 years, but not all your life
- ☐ All your life
- ☐ From surrounding area
- ☐ Just visiting

8. In the past 6 months, what types of places have you slept in? (Check all that apply)

- ☐ Own apartment / house
- ☐ Hotel / furnished room / boarding house
- ☐ Transition housing
- ☐ Hostel / shelter
- ☐ Friend's place
- ☐ Family member's place
- ☐ Camps (squatting)
- ☐ Working out of town (rigs / work camp)
- ☐ Reserve or settlement
- ☐ Couch surfing
- ☐ Detox
- ☐ Jail / prison
- ☐ Hospital
- ☐ Street (sleeping rough)
- ☐ Don't sleep (walk all night)

9. Do you currently have a home?

- ☐ Yes
- ☐ No, but I am wait-listed or have applied and am waiting to hear back
- ☐ No, and as far as I know, I have no housing application pending or am not waiting to hear back from anyone

10. How would you describe your current living situation? (Would you say …)

- ☐ Very unstable
- ☐ A little unstable
- ☐ Neither unstable nor stable
- ☐ A little stable
- ☐ Very stable

11. How satisfied are you with your current living situation? (Would you say …)
   - ☐ Very unsatisfied
   - ☐ A little unsatisfied
   - ☐ Neither unsatisfied nor satisfied
   - ☐ A little satisfied
   - ☐ Very satisfied

Now we'd like to ask you some general questions about your income. I'm interested in wherever you get your money, legal or not.

12. How much cash/money did you make in the past **30 days**? *(Includes legal and non-legal sources of income as well as assistance cheques)*
   - ☐ $0-$100
   - ☐ $100-$500
   - ☐ $500-1000
   - ☐ $1000-2000
   - ☐ $2000-3000
   - ☐ $3000 +
   - ☐ Prefer not to answer
   - ☐ Don't Know

13. In the last **30 days**, have you been legally employed? *(Does not include 'under the table' work)*
   - ☐ No
   - ☐ Yes, how many days in the last month have you worked on average? ________________
   - ☐ Don't know
   - ☐ No response

14. In the last **30 days**, have you received any assistance cheques? (Alberta Works, AISH, PDD, other)?
   - ☐ No → **Go to Q14a**
   - ☐ Yes, what kind? ____→ **Go to Q15**)
   - ☐ Don't know → **Go to Q15**
   - ☐ No response → **Go to Q15**

   a. If no, did you (or anyone acting on your behalf) try to get you set up for income assistance cheques in the past **6 months?**
      - ☐ No, no attempt to get assistance in the past 6 months
      - ☐ Yes, attempted to get assistance, but was unsuccessful
        What kind? _______________________________________
      - ☐ Yes, I applied for assistance, and I am still waiting to hear back
        What kind? _______________________________________

15. Right now, do you have any government-issued ID? (SIN, birth certificate, DL, AHC)
   - ☐ No
   - ☐ Yes
   - ☐ Don't know
   - ☐ No response

16. Right now, do you have prescription drug coverage? (e.g., you pay for only part or none of the cost of your prescriptions)

- ☐ Yes
- ☐ No
- ☐ Don't know

Now we're going to ask you a few questions about your health. Please answer according to your situation before you came to the hospital and were connected to the addiction consult team.

17. Why did you come to the hospital? ______________________________________

18. Have you ever been tested for HIV?

- ☐ Yes → **Go to Q18a**
- ☐ No → **Go to Q19**
- ☐ Don't know → **Go to Q19**
- ☐ No response → **Go to Q19**

a. When was your last HIV test?

- ☐ Within the past month
- ☐ 1 to 6 months ago
- ☐ 7 to 12 months ago
- ☐ More than 1 year but up to 2 years ago
- ☐ More than 2 years but less than 4 years ago
- ☐ 4 years ago or more
- ☐ Don't know
- ☐ No response

b. What was the result of your most recent HIV test?

- ☐ Positive for HIV—you have the virus
- ☐ Negative for HIV—you do not have the virus
- ☐ Indeterminate result
- ☐ You didn't understand the result
- ☐ You are still waiting for the result
- ☐ Your result is ready but you did not receive it yet
- ☐ Don't know
- ☐ No response

19. Have you ever been tested for Hepatitis C?

- ☐ Yes→ **Go to Q19a**
- ☐ No → **Go to Q20**
- ☐ Don't know → **Go to Q20**
- ☐ No response → **Go to Q20**

a. When was your last Hepatitis C test?

- ☐ Within the past month
- ☐ 1 to 6 months ago
- ☐ 7 to 12 months ago
- ☐ More than 1 year but up to 2 years ago
- ☐ More than 2 years but less than 4 years ago
- ☐ 4 years ago or more
- ☐ Don't know
- ☐ No response

b. What was the result of your most recent Hepatitis C test?

- ☐ Positive for Hepatitis C—you have the virus
  If positive, when were you first told you have Hepatitis C? _____years ago.
- ☐ Negative for Hepatitis C —you do not have the virus
- ☐ Indeterminate result
- ☐ You didn't understand the result
- ☐ You are still waiting for the result
- ☐ Your result is ready but you did not receive it yet
- ☐ Don't know
- ☐ No response

20. Do you currently smoke tobacco?

- ☐ Yes, I smoke tobacco everyday → **Go to Q21**
- ☐ Yes, but I don't smoke it everyday → **Go to Q22**
- ☐ Not at all → **Go to Q23**
- ☐ Don't know → **Go to Q23**
- ☐ No response → **Go to Q23**

21. If daily smoker, about how many cigarettes do you smoke each day? ____________/ per day

22. During the past **6 months**, have you tried to stop smoking?

- ☐ Yes
- ☐ No
- ☐ Don't know
- ☐ No response

23. Are you sexually active**?**

- ☐ Yes → **Go to Q23a**
- ☐ No → **Go to Q24**

a. Do you regularly use condoms during sex?

- ☐ Yes
- ☐ No
- ☐ Don't know
- ☐ No response

b. Do you regularly use contraception (condoms or otherwise)?
- ☐ Yes
- ☐ No
- ☐ Don't know
- ☐ No response

24. Over the **past two weeks**, how often have you been bothered by any of the following problems?

a. Little interest or pleasure in doing things.
- ☐ Not at all
- ☐ Several days
- ☐ More than half the days
- ☐ Nearly every day

b. Feeling down, depressed, or hopeless.
- ☐ Not at all
- ☐ Several days
- ☐ More than half the days
- ☐ Nearly every day

25. ***By placing a tick in ONE box in each group below, please indicate which statements best describe your own health state. Please answer according to your situation before you came to the hospital and were connected to the addiction consult team.***

a. **Mobility**
I have no problems in walking about ☐
I have some problems in walking about ☐
I am confined to bed ☐

b. **Self-Care**
I have no problems with self-care ☐
I have some problems washing or dressing myself ☐
I am unable to wash or dress myself ☐

c. **Usual Activities**
I have no problems with performing my usual activities ☐
I have some problems with performing my usual activities ☐
I am unable to perform my usual activities ☐

d. **Pain/Discomfort**
I have no pain or discomfort ☐
I have moderate pain or discomfort ☐
I have extreme pain or discomfort ☐

e. **Anxiety/Depression**
I am not anxious or depressed ☐
I am moderately anxious or depressed ☐
I am extremely anxious or depressed ☐

To help people say how good or bad their state of health is, we have drawn a scale (rather like a thermometer) on which the best state you can imagine is marked 100 and the worst state you can imagine is marked 0.

We would like you to indicate on this scale how good or bad your own health is, in your opinion. Please do this by drawing a line from the box below to whichever point on the scale indicates how good or bad your state of health is today.

Your own state of health

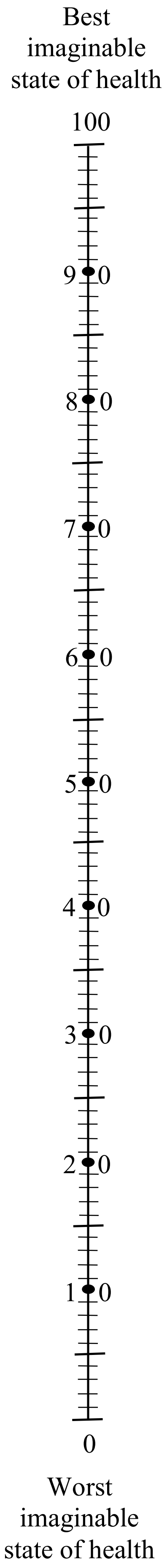

26. On a scale of 1 to 5, where 1 is 'hardly at all' and 5 is 'totally', how well organized overall would you say all your health care is?

___________________________________(1 = hardly at all, to totally = 5)

27. In general, do you feel that you yourself have to organize the health care you receive from different persons or different places?
    - ☐ No, the person who follows my case always does it for me.
    - ☐ No, the person who follows my case sometimes does it for me.
    - ☐ Yes, but it is my choice to do so.
    - ☐ Yes, I have to organize my care more than I would like.
    - ☐ Yes, I have to organize my care too much and it is too difficult.

28. Do you have a family doctor or nurse practitioner you see regularly?
    - ☐ Yes
    - ☐ No

29. Do you have a support worker in the community you work with regularly (outreach worker, social worker, housing worker, etc.)?
    - ☐ Yes
    - ☐ No

Next we'd like to ask you about your experience with the addiction consult team. Remember, team members won't know what you said, and honest feedback helps us improve the program.

30. Did the team members treating and assessing you introduce themselves?
    - ☐ Yes, all of them introduced themselves
    - ☐ Some of them introduced themselves
    - ☐ Very few or none of them introduced themselves
    - ☐ Can't remember

31. Did you have enough time to discuss your health or medical problem with the team?
    - ☐ Yes, definitely
    - ☐ Yes, to some extent
    - ☐ No

32. Did the team listen to what you had to say?
    - ☐ Yes, definitely
    - ☐ Yes, to some extent
    - ☐ No

33. Did the team explain your condition and treatment in a way you could understand?
    - ☐ Yes, definitely
    - ☐ Yes, to some extent
    - ☐ No
    - ☐ I did not need an explanation

34. If you had any anxieties or fears about your condition or treatment, did the team discuss them with you?
    - ☐ Yes, definitely
    - ☐ Yes, to some extent
    - ☐ No
    - ☐ I did not have anxieties or fears

35. Did you have confidence and trust in the doctors and nurses examining and treating you?
    - ☐ Yes, definitely
    - ☐ Yes, to some extent
    - ☐ No

36. In your opinion, did the team know enough about your condition or treatment?
    - ☐ All of them knew enough
    - ☐ Most of them knew enough
    - ☐ Only some of them knew enough
    - ☐ None of them knew enough
    - ☐ Don’t know / Can’t say

37. Did the team talk in front of you as if you weren’t there?
    - ☐ Yes, definitely
    - ☐ Yes, to some extent
    - ☐ No

Next are a couple of questions about how well you understand health information.

38. How often do you have problems learning about your medical condition because of difficulty understanding written information?
    - ☐ Never
    - ☐ Rarely
    - ☐ Sometimes
    - ☐ Often
    - ☐ Always

39. How confident are you filling out medical forms by yourself?
    - ☐ Extremely
    - ☐ Quite a bit
    - ☐ Somewhat
    - ☐ A little bit
    - ☐ Not at all

40. How often do you have someone help you read instructions, pamphlets, or other written materials from your doctor or pharmacy?
    - ☐ Never
    - ☐ Rarely
    - ☐ Sometimes
    - ☐ Often
    - ☐ Always

Here are a few questions about alcohol and drugs. Please answer as correctly and honestly as possible by indicating which answer is right for you.

****Pills and methadone do NOT count as drugs if they have been prescribed by a doctor and you take them in the prescribed dosage and by mouth.***

41. How often do you have a drink containing alcohol? (One drink means 12 oz beer, 5 ounces of wine, or 1.5 oz of hard liquor).
    - ☐ Never (Go to Q45)
    - ☐ Monthly or less
    - ☐ 2-4 times a month
    - ☐ 2-3 times a week
    - ☐ 4 or more times a week
    - ☐ Don't know
    - ☐ Refused

42. How many standard drinks containing alcohol do you have on a typical day?
    - ☐ 1 or 2
    - ☐ 3 or 4
    - ☐ 5 or 6
    - ☐ 7 to 9
    - ☐ 10 or more
    - ☐ Don't know
    - ☐ Refused

43. How often do you have six or more drinks on one occasion?
    - ☐ Never
    - ☐ Less than monthly
    - ☐ Monthly
    - ☐ Weekly
    - ☐ Daily or almost daily
    - ☐ Don't know
    - ☐ Refused

44. In the *last 6 months*, did you drink any non-beverage alcohol (e.g., cooking wine/rubbing alcohol/mouthwash/or cologne/hairspray)?
    - ☐ Yes
    - ☐ No (Go to Q45)
    - ☐ Don't know
    - ☐ Refused

    a. How often?
        - ☐ 4 or more times a week
        - ☐ 2-3 times a week
        - ☐ 2 to 4 times a month
        - ☐ Once a month or less
        - ☐ Don't know
        - ☐ Refused

45. How often do you use drugs other than alcohol? **(See list of drugs on Separate Page)**
- ☐ Never → **Go to Q48**
- ☐ Once a month or less often
- ☐ 2 – 4 times a month
- ☐ 2 – 3 times a week
- ☐ 4 times a week or more often

46. Do you use more than one type of drug on the same occasion?
- ☐ Never
- ☐ Once a month or less often
- ☐ 2 – 4 times a month
- ☐ 2 – 3 times a week
- ☐ 4 times a week or more often

47. How many times do you take drugs on a typical day when you use drugs?
- ☐ 0
- ☐ 1 – 2
- ☐ 3 – 4
- ☐ 5 – 6
- ☐ 7 or more

Now we are going to ask you about any drugs that you SMOKE, SNORT, or SWALLOW ONLY. In other words, any drugs that you use WITHOUT a needle.

48. Have you used any NON-injection drugs in the past 6 months?
- ☐ Yes
- ☐ No → **Go to Q53**
- ☐ Don't know → **Go to Q53**
- ☐ No response → **Go to Q53**

49. In the **last 6 months**, when you were using, which of the following NON-injection drugs did you use?
- ☐ Heroin (snorted or smoked)
- ☐ Morphine (pinks, greys, Kadians)
- ☐ Hydromorphone (Dilaudid, dilly's, hydros)
- ☐ Codeine (Tylenol 3/4)
- ☐ Oxycodone (Percocet, percs)
- ☐ Oxycontin (old oxys)
- ☐ OxyNeo (new oxys)
- ☐ Street Methadone (swallow)
- ☐ Street Suboxone (oral)
- ☐ Fentanyl (swallow or smoke)
- ☐ Propoxyphene (Darvon)
- ☐ Meperidine (Demerol)
- ☐ Benzodiazepines (Valium, benzos, Ativan, Restoril, Temazepam)
- ☐ Wellbutrin
- ☐ Cocaine powder (sniffed or snorted)
- ☐ Crack cocaine (smoked)
- ☐ Methamphetamines (speed, crystal meth, pint)
- ☐ Talwin (t's)
- ☐ Ritalin (r's)
- ☐ Inhalants (nitrous oxide)
- ☐ Marijuana (pot, weed)
- ☐ GHB
- ☐ Ketamine (Special K)
- ☐ MDMA (Molly)
- ☐ Mushrooms
- ☐ LSD
- ☐ Non-beverage alcohol (mouthwash, hand sanitizer, cologne, hairspray, cooking wine)
- ☐ Other:____________________________________
- ☐ Don't know
- ☐ No response

50. In the **last 6 months**, which non-injection drug did you use the most frequently?

____

51. How often did you use that drug?
- ☐ Daily
- ☐ A few times a week
- ☐ Once a week
- ☐ A few times a month
- ☐ Once a month
- ☐ Don't know
- ☐ No response

52. In the *last 6 months*, have you borrowed, lent, or shared a (crack/meth) pipe/mouthpiece?
- ☐ Yes
- ☐ No
- ☐ Don’t smoke crack or meth
- ☐ Don’t know
- ☐ No response

Now we are going to ask you about any drugs that you inject WITH a needle.

53. Have you use used any injection drugs in the past 6 months?
- ☐ Yes
- ☐ No → **Go to Q65**
- ☐ Don't know → **Go to Q65**
- ☐ No response → **Go to Q65**

54. In the last 6 months, when you were injecting, which injection drugs did you use?
- ☐ Heroin
- ☐ Speedballs (heroin/down and cocaine)
- ☐ Goofballs (heroin/down and crystal meth)
- ☐ Morphine (pinks, greys, Kadians)
- ☐ Hydromorphone (Dilaudid, dilly’s, hydros)
- ☐ Codeine (Tylenol 3/4)
- ☐ Oxycodone (Percocet, percs)
- ☐ Oxycontin (old oxys)
- ☐ OxyNeo (new oxys)
- ☐ Street Methadone
- ☐ Street Suboxone
- ☐ Fentanyl
- ☐ Propoxyphene (Darvon)
- ☐ Meperidine (Demerol)
- ☐ Benzodiazepines (Valium, benzos, Ativan, Restoril, Temazepam)
- ☐ Wellbutrin
- ☐ Cocaine powder
- ☐ Crack cocaine
- ☐ Methamphetamines (speed, crystal meth, pint)
- ☐ Other:_____________________________________
- ☐ Don’t know
- ☐ No response

55. In the **last 6 months**, which injection drug did you use the most frequently?

____

56. How often did you use that drug?
- ☐ Daily
- ☐ A few times a week
- ☐ Once a week
- ☐ A few times a month
- ☐ Once a month
- ☐ Don't know
- ☐ No response

57. In the **past 6 months**, where did you get your new rigs (needle/syringes) from? (check all that apply)
- ☐ Borrowed (not bought) on the street
- ☐ Bought on the street
- ☐ Friends/partner
- ☐ Pharmacy
- ☐ Boyle Street Community Services [Streetworks]
- ☐ Streetworks Van
- ☐ Boyle MacCauley Health Centre
- ☐ HIV Edmonton
- ☐ George Spady Centre
- ☐ STI Clinic
- ☐ Other (please specify): ____

58. In the last 6 months, what percentage of your new rigs came from a needle exchange program?
- ☐ None
- ☐ Not many (less than 25%)
- ☐ Some (25%-75%)
- ☐ Most (more than 75%)
- ☐ All (100%) → **Go to Q60**

59. Do you ever find it hard to get new rigs when you need them?
- ☐ Yes → **Go to Q59a**
- ☐ Sometimes → **Go to Q59a**
- ☐ No → **Go to Q60**

   a. If yes or sometimes, why do you find it hard to get new rigs? (check all that apply)
   - ☐ Out of the needle exchange area
   - ☐ Hard to find new needles at night
   - ☐ Don't like going to the needle exchange
   - ☐ Too drug sick or high
   - ☐ Missed the needle exchange van
   - ☐ Refused by pharmacy or store
   - ☐ Needle exchange hours of operation
   - ☐ Incarcerated

60. In the **last 6 months**, if you wanted to fix but didn't have a new rig, what did you do? (check all that apply)

- ☐ Reuse one of my old ones
- ☐ Use a rig that is not my own
- ☐ Clean it
- ☐ Don't clean it
- ☐ Go to the needle exchange
- ☐ Buy one
- ☐ Use the drugs a different way
- ☐ Borrow a rig from someone I know
- ☐ Steal one
- ☐ I don't fix if I don't have a new rig/always have a new rig

61. In the **last 6 months**, did you fix with **a rig** (or other equipment such as cookers, filters, etc.) that had already been used by someone else?

- ☐ Yes
- ☐ No

62. In the last 6 months did you lend a used rig to someone else?

- ☐ Yes
- ☐ No
- ☐ Don't know
- ☐ Refused

63. Has a healthcare provider, such as a nurse or someone else, ever shown you how to inject safely?

- ☐ Yes
- ☐ No

64. In the **last 6 months,** how often did you inject drugs in public places (washrooms, shelter, outside, alley, river valley, etc., NOT at a private residence)?

- ☐ Always (100% of the time)
- ☐ Usually (more than 75% of the time)
- ☐ Sometimes (26% to 74% of the time)
- ☐ Occasionally (less than 25% of the time)
- ☐ Never

65. In the last **6 months**, did you go on any runs or binges (that is, when you use non-injection or injection drugs more than usual)?

- ☐ No
- ☐ Yes, how often did you binge? _______________/month OR _______/6 months
- ☐ Don't know
- ☐ No response

66. In the last **6 months** have you overdosed by accident (i.e., where you had a negative reaction from using too much drugs)?
   - ☐ Yes
   - ☐ No
   - ☐ Don't know
   - ☐ No response

67. Have you ever been given take-home Narcan/Naloxone and trained on how to use it on someone if they overdose?
   - ☐ No
   - ☐ Yes **→Go to Q68**
   - ☐ Don't know **→Go to Q68**
   - ☐ No response **→Go to Q68**

   a. If no, why not? [Check one]
      - ☐ Didn't know it was available/allowed
      - ☐ Never been offered
      - ☐ Not interested
      - ☐ Don't feel comfortable using it
      - ☐ Don't use opiates or down
      - ☐ Other:____________________
      - ☐ Don't know
      - ☐ No response

Now we are going to ask you some questions about your addiction and mental health status and some of the services you may have used recently. We are asking about how things were BEFORE you came to the hospital.

68. Before you came to the hospital, had a health professional ever told you that you have an addiction?
   - ☐ Yes, in the past 12 months
   - ☐ Yes, but not in the past 12 months
   - ☐ Yes (don't know when)
   - ☐ No
   - ☐ Don't know
   - ☐ No response

69. Has a health professional ever told you that you have a mental disorder?
   - ☐ Yes, in the past 12 months
   - ☐ Yes, but not in the past 12 months
   - ☐ Yes (don't know when)
   - ☐ No
   - ☐ Don't know
   - ☐ No response

70. Do you think you have ever had an addiction problem that has not been diagnosed by a professional? *(By addiction problem I mean misuse of things like alcohol, street drugs, or prescription medications to get high, or engaging in behaviours like gambling, sex, or work in a way that creates problems in life*)?

☐ Yes, in the past 12 months
☐ Yes, but not in the past 12 months
☐ Yes (don't know when)
☐ No
☐ Don't know
☐ No response

71. Do you think you have ever had a mental health problem that has not been diagnosed by a professional?

☐ Yes, in the past 12 months
☐ Yes, but not in the past 12 months
☐ Yes (don't know when)
☐ No
☐ Don't know
☐ No response

72. In the past 12 months before you came to the hospital this time, did you receive information (about treatments or available services) because of problems with your emotions, mental health, or use of alcohol or drugs? (Check ONE only).

☐ Yes, in the past 12 months → **Go to Q72a**
☐ No, but I think I needed this kind of help in the past 12 months → **Go to Q72b**
☐ No, I did not need this kind of help in the past 12 months → **Go to Q73**
☐ Don't know → **Go to Q73**
☐ No response → **Go to Q73**

a. Do you think you got as much information as you needed?

☐ No → **Go to Q72b**
☐ Yes → **Go to Q73**
☐ Don't know → **Go to Q73**
☐ No response → **Go to Q73**

b. Please indicate if each of the following reasons stopped you from getting any or enough help in the past 12 months before you came to the hospital (Check all that apply).

- ☐ I preferred to manage myself
- ☐ I didn't know where to get help
- ☐ I was afraid to ask for help or what others would think of me
- ☐ I couldn't afford the money
- ☐ I asked but I didn't get help
- ☐ I didn't think anything would help/nothing can help me
- ☐ I don't want to get help at this time
- ☐ Wait list was too long/no spaces are available
- ☐ I was only allowed a limited amount of help [information]
- ☐ Other (please specify):____
- ☐ Don't know
- ☐ No response

73. In the past 12 months, before you came to the hospital this time, did you receive medication (or tablets) because of problems with your emotions, mental health, or use of alcohol or drugs? *(e.g., methadone, antidepressants, benzodiazepines etc.)* (Check ONE).

- ☐ Yes, in the past 12 months → **Go to Q73a**
- ☐ No, but I think I needed this kind of help in the past 12 months → **Go to Q73b**
- ☐ No, I did not need this kind of help in the past 12 months → **Go to Q74**
- ☐ Don't know → **Go to Q74**
- ☐ No response → **Go to Q74**

a. Do you think you got as much medication as you needed?

- ☐ No → **Go to Q73b**
- ☐ Yes → **Go to question Q74**
- ☐ Don't know → **Go to Q74**
- ☐ No response → **Go to Q74**

b. Please indicate if each of the following reasons stopped you from getting any or enough help in the past 12 months (Check all that apply).

- ☐ I preferred to manage myself
- ☐ I didn't know where to get help
- ☐ I was afraid to ask for help or what others would think of me
- ☐ I couldn't afford the money
- ☐ I asked but I didn't get help
- ☐ I didn't think anything would help/nothing can help me
- ☐ I don't want to get help at this time
- ☐ Wait list was too long/no spaces are available
- ☐ I was only allowed a limited amount of help [medication]
- ☐ Other (please specify): ______________________________________
- ☐ Don't know
- ☐ No response

74. In the past 12 months, before you came to the hospital this time, did you receive hospital care (overnight or longer) because of problems with your emotions, mental health, or use of alcohol or drugs? (e.g., treating an infection or abscess, overdose, psychosis, etc.) (Check ONE).

- ☐ Yes, in the past 12 months → **Go to Q74a**
- ☐ No, but I think I needed this kind of help in the past 12 months → **Go to Q74b**
- ☐ No, I did not need this kind of help in the past 12 months → **Go to question Q75**
- ☐ Don't know → **Go to Q75**
- ☐ No response → **Go to Q75**

a. Do you think you got as much hospital care as you needed?

- ☐ No → **Go to Q74b**
- ☐ Yes → **Go to question Q75**
- ☐ Don't know → **Go to Q75**
- ☐ No response → **Go to Q75**

b. Please indicate if each of the following reasons stopped you from getting any or enough help in the past 12 months (Check all that apply).

- ☐ I preferred to manage myself
- ☐ I didn't know where to get help
- ☐ I was afraid to ask for help or what others would think of me
- ☐ I couldn't afford the money
- ☐ I asked but I didn't get help
- ☐ I didn't think anything would help/nothing can help me
- ☐ I don't want to get help at this time
- ☐ Wait list was too long/no spaces are available
- ☐ I was only allowed a limited amount of help [hospital care]
- ☐ Other (please specify): ____
- ☐ Don't know
- ☐ No response

75. In the past 12 months, before you came to the hospital this time, did you receive counselling (outside of a hospital including any kind of help to talk through your problems) because of problems with your emotions, mental health, or use of alcohol or drugs? (Check ONE).

- ☐ Yes, in the past 12 months → **Go to Q75a**
- ☐ No, but I think I needed this kind of help in the past 12 months → **Go to Q75b**
- ☐ No, I did not need this kind of help in the past 12 months → **Go to Q76**
- ☐ Don't know → **Go to Q76**
- ☐ No response → **Go to Q76**

a. Do you think you got as much counselling as you needed?

- ☐ No → **Go to Q75b**
- ☐ Yes → **Go to Q76**
- ☐ Don't know → **Go to Q76**
- ☐ No response → **Go to Q76**

b. Please indicate if each of the following reasons stopped you from getting any or enough help in the past 12 months (Check all that apply).

- ☐ I preferred to manage myself
- ☐ I didn't know where to get help
- ☐ I was afraid to ask for help or what others would think of me
- ☐ I couldn't afford the money
- ☐ I asked but I didn't get help
- ☐ I didn't think anything would help/nothing can help me
- ☐ I don't want to get help at this time
- ☐ Wait list was too long/no spaces are available
- ☐ I was only allowed a limited amount of help [counselling]
- ☐ Other (please specify): ____
- ☐ Don't know
- ☐ No response

76. In the past 12 months, before you came to the hospital this time, did you receive social interventions (to help sort out practical issues such as housing or money problems) because of problems with your emotions, mental health, or use of alcohol or drugs? (e.g., income support, shelters, housing, etc.) (Check ONE).

- ☐ Yes, in the past 12 months → **Go to Q76a**
- ☐ No, but I think I needed this kind of help in the past 12 months → **Go to Q76b**
- ☐ No, I did not need this kind of help in the past 12 months → **Go to question Q77**
- ☐ Don't know → **Go to Q77**
- ☐ No response → **Go to Q77**

a. Do you think you got as much social interventions as you needed?

- ☐ No → **Go to Q76b**
- ☐ Yes → **Go to Q77**
- ☐ Don't know
- ☐ No response

b. Please indicate if each of the following reasons stopped you from getting any or enough help in the past 12 months (Check all that apply).

- ☐ I preferred to manage myself
- ☐ I didn't know where to get help
- ☐ I was afraid to ask for help or what others would think of me
- ☐ I couldn't afford the money
- ☐ I asked but I didn't get help
- ☐ I didn't think anything would help/nothing can help me
- ☐ I don't want to get help at this time
- ☐ Wait list was too long/no spaces are available
- ☐ I was only allowed a limited amount of help [social interventions/supports]
- ☐ Other (please specify): ____
- ☐ Don't know
- ☐ No response

77. In the past 12 months, before you came to the hospital this time, did you receive skills training (help to improve your ability to work, care for yourself, use your time or to meet people) because of problems with your emotions, mental health, or use of alcohol or drugs? (Check ONE).

- ☐ Yes, in the past 12 months → **Go to Q77a**
- ☐ No, but I think I needed this kind of help in the past 12 months → **Go to Q77b**
- ☐ No, I did not need this kind of help in the past 12 months → **Go to Q78**
- ☐ Don't know → **Go to Q78**
- ☐ No response → **Go to Q78**

a. Do you think you got as much skills training as you needed?

- ☐ No → **Go to Q77b**
- ☐ Yes → **Go to Q78**
- ☐ Don't know
- ☐ No response

b. Please indicate if each of the following reasons stopped you from getting any or enough help in the past 12 months (Check all that apply).

- ☐ I preferred to manage myself
- ☐ I didn't know where to get help
- ☐ I was afraid to ask for help or what others would think of me
- ☐ I couldn't afford the money
- ☐ I asked but I didn't get help
- ☐ I didn't think anything would help/nothing can help me
- ☐ I don't want to get help at this time
- ☐ Wait list was too long/no spaces are available
- ☐ I was only allowed a limited amount of help [skills training]
- ☐ Other (please specify): ____
- ☐ Don't know

78. In the past 12 months, before you came to the hospital this time, did you received access to harm reduction (services [like needle exchange or overdose prevention] to reduce the risk of harm related to using drugs) because of problems with your use of alcohol or drugs? (Check ONE).

- ☐ Yes, in the past 12 months → **Go to Q78a**
- ☐ No, but I think I needed this kind of help in the past 12 months → **Go to Q78b**
- ☐ No, I did not need this kind of help in the past 12 months → **Go to Q79**
- ☐ Don't know → **Go to Q79**
- ☐ No response → **Go to Q79**

a. Do you think you got as much harm reduction services as you needed?

- ☐ No → **Go to Q78b**
- ☐ Yes → **Go to Q79**
- ☐ Don't know→ **Go to Q79**
- ☐ No response→ **Go to Q79**

b. Please indicate if each of the following reasons stopped you from getting any or enough help in the past 12 months (Check all that apply).

- ☐ I preferred to manage myself
- ☐ I didn't know where to get help
- ☐ I was afraid to ask for help or what others would think of me
- ☐ I couldn't afford the money
- ☐ I asked but I didn't get help
- ☐ I didn't think anything would help/nothing can help me
- ☐ I don't want to get help at this time
- ☐ Wait list was too long/no spaces are available
- ☐ I was only allowed a limited amount of help [harm reduction]
- ☐ Other (please specify): ____
- ☐ Don't know

79. Have you ever been in a substance use treatment program such as detox, AA, NA, CA, in-patient treatment, recovery house, etc.? (*this does not include methadone or suboxone*)

- ☐ Yes, in the past 12 months→ **Go to Q79a**
- ☐ Yes, but not in the past 12 months→ **Go to Q79a**
- ☐ No → **Go to Q80**
- ☐ Don't know → **Go to Q80**
- ☐ No response → **Go to Q80**

a. If yes, how many times have you been in treatment before?

- ☐ 1 time
- ☐ 2-3 times
- ☐ 4-5 times
- ☐ 5-8 times
- ☐ 8 or more times
- ☐ Don't know
- ☐ No response

b. What kinds of treatment programs did you attend?

- ☐ Detox/youth detox
- ☐ Daytox (outpatient detox)
- ☐ Recovery house
- ☐ Treatment centre
- ☐ Sessions with a counsellor
- ☐ NA, CA, AA, SMART or other self-help group
- ☐ In-patient or residential treatment
- ☐ Out-patient treatment (includes day, evening, and weekend programs)
- ☐ Drug treatment court
- ☐ Other (please specify): _______________________________________
- ☐ Don't know
- ☐ No response

80. Have you ever been on methadone or suboxone for problems with opiates [down]?

- ☐ Yes → **Go to Q80a**
- ☐ No → **Go to Q80c**
- ☐ Don't know → **Go to Q81**
- ☐ No response → **Go to Q81**

a. Were you on a methadone/suboxone treatment program just before you came to the hospital?

- ☐ No → **Go to Q80b**
- ☐ Yes → **Go to Q81**
- ☐ Don't know → **Go to Q81**
- ☐ No response → **Go to Q81**

b. Why did you stop taking methadone/suboxone?

- ☐ Didn't want to take it anymore
- ☐ Side effects
- ☐ Could not get to pharmacy
- ☐ Didn't comply with the program and taken off by my doctor
- ☐ Went to jail
- ☐ Other (please specify):________________________
- ☐ Don't know
- ☐ No response

→ Go to Q81

c. If you've never been on methadone/suboxone, why not? [Check all that apply].

- ☐ Waiting list
- ☐ Don't know about it or any programs
- ☐ Turned down by a program/doctor
- ☐ No program nearby
- ☐ Not interested
- ☐ Too inconvenient
- ☐ Don't want to stop using drugs
- ☐ Don't trust methadone, worried about side effects
- ☐ Prefer abstinence/not a solution
- ☐ I have no problems with opiates [down]
- ☐ Don't know
- ☐ No response

81. Have you received mental health care in the past 6 months from a psychologist, psychiatrist, counsellor or other trained mental health professional?

- ☐ Yes
- ☐ No
- ☐ Don't know
- ☐ No response

Next, a few questions about your experience with the justice system. We are asking these questions because sometimes people who experience alcohol or drug problems end up involved with the criminal justice system, remember that your answers are confidential.

82. What is your current legal status? ***(Check all that apply)***

- ☐ Under no judicial restraint (None of the options below apply)
- ☐ On parole
- ☐ On probation
- ☐ Serving a conditional or community sentence
- ☐ Under bail, pending charges, or warrant
- ☐ Fines
- ☐ Other, please specify: ____

83. In the last 6 months, have you been attacked or assaulted (including sexual assault), or suffered any kind of violence?

- ☐ Yes: How many times has this happened? ______________________________
- ☐ No
- ☐ Don't know
- ☐ Refused

84. In the last 6 months, have you been the **victim** of a theft, robbery, fraud or other kind of property crime?

- ☐ Yes: How many times has this happened? ________________________
- ☐ No
- ☐ Don't know
- ☐ Refused

85. In the last **6 months**, have you been involved in any of the following activities?

Activity	# of occurrences
☐ Break and enter	____________
☐ Theft under $5000	____________
☐ Theft over $5000	____________
☐ Breached probation	____________
☐ Failed to appear	____________
☐ Possession of stolen property	____________
☐ Possession of drugs for the purpose of trafficking/dealing	____________
☐ Robbery	____________
☐ Shoplifting	____________
☐ Assault	____________
☐ Mischief/disorderly conduct	____________
☐ Other (please specify):____________________	____________

The next section is going to ask about how you feel others have treated you.

86. Have you ever experienced discrimination, been prevented from doing something, or been hassled or made to feel inferior in any of the following situations because of your race, ethnicity or colour?

    a. At school?
    - ☐ Never
    - ☐ Once
    - ☐ 2-3 times
    - ☐ 4 or more times

    b. Getting hired or getting a job?
    - ☐ Never
    - ☐ Once
    - ☐ 2-3 times
    - ☐ 4 or more times

    c. At work?
    - ☐ Never
    - ☐ Once
    - ☐ 2-3 times
    - ☐ 4 or more times

    d. Getting housing?
    - ☐ Never
    - ☐ Once
    - ☐ 2-3 times
    - ☐ 4 or more times

    e. Getting medical care?
    - ☐ Never
    - ☐ Once
    - ☐ 2-3 times
    - ☐ 4 or more times

    f. Getting service in a store or restaurant?
    - ☐ Never
    - ☐ Once
    - ☐ 2-3 times
    - ☐ 4 or more times

    g. Getting financial advice or help?
    - ☐ Never
    - ☐ Once
    - ☐ 2-3 times
    - ☐ 4 or more times

h. On the street or in a public setting?

- ☐ Never
- ☐ Once
- ☐ 2-3 times
- ☐ 4 or more times

i. From the police or in the courts?

- ☐ Never
- ☐ Once
- ☐ 2-3 times
- ☐ 4 or more times

Next, we would like to learn more about things that might have happened while you were growing up. This is so we can understand our patients and make sure we have the right kind of program to help. You don't have to answer any or all of these questions. Let us know if you would like to skip this section.

As a child, did you ever witness abuse in your household (physical, sexual, emotional)?

- ☐ Yes
- ☐ No

As a child, did you ever experience abuse (physical, sexual, emotional)?

- ☐ Yes
- ☐ No

Did you ever spend time in foster care?

- ☐ Yes
- ☐ No

Did you ever spend time in a residential school?

- ☐ Yes
- ☐ No
